# HBX-induced miR-5188 impairs FOXO1 to stimulate β-catenin nuclear translocation and promotes tumor stemness in hepatocellular carcinoma

**DOI:** 10.7150/thno.37717

**Published:** 2019-10-12

**Authors:** Xian Lin, Shi Zuo, Rongcheng Luo, Yonghao Li, Guifang Yu, Yujiao Zou, Yan Zhou, Zhan Liu, Yiyi Liu, Yingying Hu, Yingying Xie, Weiyi Fang, Zhen Liu

**Affiliations:** 1 Affiliated Cancer Hospital & Institute of Guangzhou Medical University; Laboratory of Protein Modification and Degradation, State Key Laboratory of Respiratory Disease, Guangzhou Medical University, Guangzhou, China.; 2 Cancer Institute, School of Basic Medical Science, Southern Medical University, Guangzhou, Guangdong, China.; 3 Cancer Center, Integrated Hospital of Traditional Chinese Medicine, Southern Medical University, Guangzhou, Guangdong, China.; 4 The Affiliated Hospital of Guizhou Medical University, Guiyang, Guizhou, China.; 5 Department of Oncology, The Fifth Affiliated Hospital of Guangzhou Medical University, Guangzhou, Guangdong, China.; 6 Brain Hospital of Hunan Province, Changsha, Hunan, China.; 7 Department of Gastroenterology, Hunan People's Hospital, Changsha, Hunan, China.

**Keywords:** MiR-5188, Hepatitis X protein, Cancer stem cells, Hepatocellular carcinoma

## Abstract

Cancer stem cells (CSCs) are the key factor in determining cancer recurrence, metastasis, chemoresistance and patient prognosis in hepatocellular carcinoma (HCC). The role of miR-5188 in cancer stemness has never been documented. In this study, we investigated the clinical and biological roles of miR-5188 in HCC.** Methods:** MiRNA expression in HCC was analyzed by bioinformatics analysis and *in situ* hybridization. The biological effect of miR-5188 was demonstrated in both *in vitro* and *in vivo* studies through the ectopic expression of miR-5188. The target gene and molecular pathway of miR-5188 were characterized using bioinformatics tools, dual-luciferase reporter assays, gene knockdown, and rescue experiments. **Results:** MiR-5188 was shown to be upregulated and confer poor prognosis in HCC patient data from TCGA database. MiR-5188 was subsequently identified as a significant inducer of cancer stemness that promotes HCC pathogenesis. Specifically, the targeting of miR-5188 by its antagomir markedly prolonged the survival time of HCC-bearing mice and improved HCC cell chemosensitivity *in vivo*. Mechanistic analysis indicated that miR-5188 directly targets FOXO1, which interacts with β-catenin in the cytoplasm to reduce the nuclear translocation of β-catenin and promotes the activation of Wnt signaling and downstream tumor stemness, EMT, and c-Jun. Moreover, c-Jun transcriptionally activates miR-5188 expression, forming a positive feedback loop. Interestingly, the miR-5188-FOXO1/β-catenin-c-Jun feedback loop was induced by hepatitis X protein (HBX) through Wnt signaling and participated in the HBX-induced pathogenesis of HCC. Finally, analyses of transcriptomics data and our clinical data supported the significance of the abnormal expression of the miR-5188 pathway in HCC pathogenesis.** Conclusions:** These findings present the inhibition of miR-5188 as a novel strategy for the efficient elimination of CSCs to prevent tumor metastasis, recurrence and chemoresistance in patients with hepatocellular carcinoma. Our study highlights the importance of miR-5188 as a tumor stemness inducer that acts as a potential target for HCC treatment.

## Introduction

Cancer stem cells (CSCs) are responsible for maintaining tumors by manipulating genetic and nongenetic factors to promote metastasis, resist therapies, sustain the tumor microenvironment and support regrowth of the tumor, resulting in recurrence 1-3. Studies of the key traits and mechanisms of CSCs in determining recurrence, metastasis, and therapeutic resistance provide opportunities to develop new therapeutic treatments and improve patient outcomes.

The present evidence supports that a subset of causes of hepatocellular carcinoma (HCC) progression reside outside of the protein coding genes, with miRNAs being one area that has drawn increasing attention 4. Previous studies suggest that miRNAs are capable of regulating cancer stemness in HCC 5-7. However, the biological role of miR-5188 in tumors, including HCC, remains undefined.

Hepatitis B virus is a major etiologic agent of HCC, and HBV-encoded X protein (HBX), an HCC stemness inducer, has been identified as an essential component of Wnt/β-catenin signaling activation 8-10. Activation of Wnt signaling contributes to enhancing the CSC properties of HCC 11-12. However, whether HBX can regulate miRNAs to regulate HCC stemness is not fully understood.

Here, we report a miR-5188-FOXO1/β-catenin-c-Jun positive feedback loop that promotes cell stemness, metastasis, proliferation, and chemoresistance in HCC. Interestingly, this loop is facilitated by HBX and further activates β-catenin/c-Jun signaling. Collectively, our results suggest that targeting miR-5188 delays HCC progression and that miR-5188 is an alternative strategy for HCC treatment.

## Methods

### Cell culture

Bel-7402, Bel-7404, HepG2, SMMC-7721, QGY-7703, Hep3B, Huh7, MHCC97L, MHCC97H, PLC/PRF/5 and HCCLM3 cells were purchased from the Shanghai Cell Bank of the Chinese Academy of Sciences in China. LO2 and HEK293T cells were obtained from the Cancer Research Institute of Southern Medical University in Guangzhou, China. Cells were propagated in DMEM supplemented with 10% (v/v) fetal bovine serum (Biowest, Loire Valley, France) at 37 °C in a humidified atmosphere of 5% CO2 in an incubator.

### Cell transfection

Plasmids were purchased from Vigene Biosciences (Shangdong, China). siRNAs, mimics and inhibitors were designed and synthesized by Guangzhou RiboBio Co., Ltd. (Guangzhou, China) (Table S1). Exponentially growing cells were seeded in a cell culture plate or dish (NEST Biotech Co., Ltd., China) before transfection. Plasmids, siRNAs, mimics and inhibitors were then transfected into cells using Lipofectamine TM 2000 (Invitrogen Biotechnology Co., Ltd., Shanghai, China) according to the manufacturer's protocol. Cells were collected 48-72 h after transfection for further experiments.

### Lentivirus production and infection

Lentiviral particles encoding hsa-miR-5188 precursor (miR-5188) were designed and constructed by GeneChem (Shanghai, China) (Table S1). Cells were infected with lentiviral vector, and the expression of miR-5188 was detected by QPCR.

### RT-PCR and QPCR

Total RNA was isolated from cells or harvested tissues. cDNA was synthesized using reverse transcription reagents (TaKaRa Bio, Inc., Shiga, Japan), and cDNA was used as a template for amplification using specific primers (Table S2). The Bio-Rad T100 and Bio-Rad CFX96 detection systems were applied for RT-PCR and QPCR, respectively, according to the manufacturer's instructions.

### Western blot analysis

Cell lysates were obtained in lysis buffer, and protein concentrations were determined using a BCA protein assay kit (Thermo Scientific, Waltham, MA, USA). Proteins were separated by SDS-PAGE and transferred onto polyvinyl difluoride membranes, which were immunoprobed with the corresponding antibodies. The proteins were detected using enhanced chemiluminescence reagent (Thermo Scientific, Waltham, MA, USA). Antibodies against the following proteins were used: HBX, β-catenin, nonphospho (active) β-catenin, c-Jun, c-Myc, CCND1, CD44, Slug, SOX2, OCT4, Nanog, ABCG2, ABCB1, E-cadherin, N-cadherin, vimentin, FOXO1, GAPDH, histone and β-actin. The dilutions and sources of the antibodies are shown in Table S3. Images were captured using a ChemiDocTM CRS+ Molecular Imager (Bio-Rad, Hercules, CA, USA).

### Hepatosphere formation assay

Cells separated in single cell suspensions were seeded in 6-well ultra-low attachment plates (Corning, Inc., NY, USA) at a density of 5×10^3^ cells/well and cultured in serum-free DMEM/F12 with FGF (20 ng/ml), EGF (20 ng/ml) and B27 (2%). After incubation for 2-3 weeks, tumor spheres were photographed under a microscope and then separated into single cells to form new tumor spheres. The size and number of tumor spheres were analyzed after continuous passaging for three generations.

### Flow cytometry analysis

To determine the proportion of CD44^high^CD133^high^ cells, 1×10^6^ cells were separated into single cells and resuspended in 200 µl of PBS containing fluorescein isothiocyanate (FITC)-conjugated anti-CD44 and phycoerythrin (APC)-conjugated anti-CD133 antibodies. After incubation with the antibodies for 30 min on ice, the cells were rinsed with PBS and analyzed by flow cytometry. For side population analysis, 1×10^6^ cells were resuspended in DMEM containing 2% FBS and treated with 5 μg/ml Hoechst 33342 (Sigma-Aldrich, MO, USA) for 90 min at 37 °C with gentle blending every 10 min. Samples simultaneously incubated with 50 μmol/L verapamil (Sigma-Aldrich, MO, USA) served as a negative control. Cells were washed with ice-cold PBS and then subjected to flow cytometry. Propidium iodide was used to identify dead cells.

### Migration, invasion and wound healing assays

A Transwell (BD Biosciences, NJ, USA) assay was performed to detect cell migration and invasion abilities. Cells were suspended in 100 μl of DMEM without serum and seeded into the top chamber of Transwell inserts coated with Matrigel (BD Biosciences, NJ, USA) or left uncoated, and the bottom chambers were filled with 500 μl of DMEM supplemented with 10% FBS. The migrated cells were stained with crystal violet and then photographed and quantified by counting the cell numbers in five random fields. Cells were seeded in 6-well plates for growth into a confluent monolayer, and scratches were created using a pipette tip. The progression of cell migration was photographed at initiation and 24, 48 and 72 h after wounding.

### MTT assay

Cell proliferation and drug sensitivity were determined using the MTT assay. Exponentially growing cells were seeded into 96-well plates in medium and incubated overnight to allow cell adherence. Cell viability was measured using MTT (5 mg/ ml) (Sigma-Aldrich, MO, USA).

### Colony formation assay

Cells were plated in 6-well plates at a density of 200 cells per well. The medium was refreshed after 24 h of incubation. After 14 days of culture, colonies were stained with a hematoxylin solution after fixation with methanol for 15 min and finally counted under a microscope.

### EdU incorporation assay

EdU incorporation was assessed using an Apollo567 *In Vitro* Imaging Kit (RiboBio Co., Ltd., Guangzhou, China) according to the manufacturer's protocol. Cells were incubated with 10 μM EdU for 2 h and then fixed with 4% paraformaldehyde. After permeabilization with 0.3% Triton X-100, the cells were stained with Apollo fluorescent dyes and 5 μg/ml DAPI.

### Immunofluorescence and confocal microscopy

Cells were plated on coverslips in a 48-well plate and cultured overnight to allow for cell adherence. After fixation with 4% paraformaldehyde and permeabilization in 0.2% Triton X-100, the cells were incubated with antibodies. They were then counterstained with 0.2 mg/ml DAPI and visualized using a fluorescence confocal microscope (Carl Zeiss LSM800).

### *In vivo* tumor xenograft study

The protocols for animal experiments complied with the requirements of the Institutional Animal Ethical Committee, Experimental Animal Center of Southern Medical University, China, and followed international guidelines for proper animal care and maintenance. A subcutaneous xenograft mouse model in which to detect the tumor formation was established as previously described 13. A series of 1×10^6^, 5×10^5^, 1×10^5^ and 5×10^4^ cells were inoculated into the flanks of 4- to 5-week-old male BALB/c-nu mice (N=6 per group). A subcutaneous xenograft mouse model in which 5×10^6^ cells were suspended in 0.1 ml of PBS and subcutaneously injected into mice (N=5 per group) was also adopted to evaluate tumor growth. The mice were sacrificed, and tumors were excised, weighed, and processed for further experiments. Tumor volume was calculated as maximum diameter (L) × minimal diameter (W)^2^/2 14. Additionally, a subcutaneous xenograft mouse model to assess the chemoresistance of the tumor was developed. Four- to five-week-old male BALB/c-nu mice (N=10 per group) were subcutaneously inoculated in the flank with 5×10^6^ cells. The mice were then intraperitoneally treated with epirubicin at 5 mg/kg of body weight a total of 4 times on days 7, 11, 15 and 19 after cell inoculation 15; intraperitoneally treated with cisplatin at 3 mg/kg or 5-fluorouracil at 30 mg/kg three times weekly for 2 weeks 16; or intratumorally injected with 10 nmol of miR-5188 antagomir (RiboBio Co. Ltd., Guangzhou, China) in 0.1 ml of normal saline every 3 days for two weeks 17. Survival curves were analyzed using Kaplan-Meier analysis.

An orthotopic tumor model and a pulmonary metastasis model were applied for *in vivo* metastasis assays. A total of 5×10^6^ cells were injected under the liver capsule (N=7 per group) or intravenously inoculated into the tail veins of the mice (N=5 per group). Optical and pathological images were collected to visualize primary tumor growth and metastatic lesion formation.

### Luciferase reporter assays

A fragment of the FOXO1 3'-UTR (wild-type 3'-UTR) was amplified. Site-directed mutagenesis (mut) of the miR-5188-binding site was conducted using the GeneTailor Site-Directed Mutagenesis System (Invitrogen, Guangzhou, China). The wt 3'-UTR or mut 3'-UTR were cloned into the psiCHECK-2 vector for luciferase reporter assays. The vector was cotransfected with miR-5188 mimics/inhibitor or the control sequence into cells, and luciferase activity was measured 48 h after transfection using the Dual-Luciferase Reporter Assay System (Promega Corporation, Madison, WI, USA). To investigate the effect of c-Jun on the transcriptional activity of miR-5188, fragments encoding c-Jun-binding sites were cloned into the pGL4.1-Basic luciferase reporter vector, and vectors containing mutant c-Jun-binding sites were also constructed. These vectors and the c-Jun plasmid were cotransfected into cells, following which luciferase activity was detected.

### TOP/FOP luciferase reporter assay

Transcriptional activity assays were conducted using the Luciferase Assay System according to the manufacturer's instructions as previously described 18. Briefly, cells were cotransfected with TOPflash or FOPflash with pRL (Millipore Corporation, Billerica, MA, USA) using Lipofectamine 2000. Twenty-four hours after transfection, cells were lysed, and luciferase activity was measured using a Dual-Luciferase Reporter Assay System (Promega Corporation, Madison, WI, USA) on a luminometer (BioTek Instruments, Winooski, VT, USA). The transcriptional activity of each sample is presented as the ratio of firefly luciferase activity to Renilla luciferase activity.

### RNA immunoprecipitation (RIP) assay

RNA immunoprecipitation assays were performed using an RIP assay kit (Millipore Corp., Billerica, MA, USA). According to the manufacturer's protocol, the protein-RNA complex was isolated, and anti-AGO2 or IgG was added to the reaction system for immunoprecipitation. After RNA purification, the immunoprecipitated RNA was subjected to QPCR and/or PCR. IgG served as a negative control.

### Chromatin immunoprecipitation (ChIP) assay

Chromatin immunoprecipitation assays were performed using a ChIP assay kit (Thermo Scientific, Waltham, MA, USA). According to the manufacturer's protocol, chromatin was crosslinked, isolated, and digested with micrococcal nuclease to obtain DNA fragments. Anti-c-Jun or IgG was added to the reaction system for immunoprecipitation. After elution and purification, the recovered DNA fragments were subjected to QPCR and/or PCR. IgG served as a negative control.

### Electrophoretic mobility shift assay (EMSA)

An electrophoretic mobility shift assay was conducted using an EMSA Kit (BersinBio, Guangzhou, China) according to the manufacturer's instructions. Nuclear extracts were obtained from cells, and their concentrations were determined using a BCA assay kit. An EMSA was performed with a reaction mixture containing nuclear extracts and biotin-labeled probes. Competition or supershift assays were performed by adding a 100-fold excess of cold competitors (unlabeled wild-type or mutant probes) (Table S4) or polyclonal rabbit anti-c-Jun (Cell Signaling Technology) to the reaction mixture. After electrophoresis and incubation, signals were recorded and analyzed.

### Coimmunoprecipitation (Co-IP)

Co-IP was performed using a Pierce Co-Immunoprecipitation kit (Thermo Scientific, Waltham, MA, USA) according to the manufacturer's instructions. Briefly, total proteins were extracted from cells, and their concentration was quantified. A total of 5 mg of protein was incubated with 10 μg of specific antibodies or IgG overnight at 4°C. After elution, the recovered proteins were subjected to Western blot analysis. IgG was used as a negative control.

### Cell fractionation assay

A cell fractionation assay was conducted using an NE-PER@ Nuclear and Cytoplasmic Extraction kit (Thermo Scientific Pierce, UK) according to the manufacturer's instructions. Briefly, cells were harvested, washed with PBS by pipetting and then incubated with ice-cold CER I for 10 min at 4°C. After incubation, CER II extraction reagent was added to the reaction mixture for another 1 minute, and the lysate was centrifuged at 16000 ×g for 5 min. The supernatant (cytoplasmic extract) was carefully transferred into a fresh microcentrifuge tube and stored on ice. The pellet was resuspended in NER extraction regent and incubated for 40 min on ice. The suspension was then centrifuged at 16000 ×g for 10 min, and the supernatant (nuclear extract) was transferred to a fresh microcentrifuge tube and stored on ice. The proteins were quantitated using a BCA protein assay kit and further analyzed by Western blot analysis.

### Tissue specimens

One hundred and eighty-five (185) paraffin-embedded HCC specimens and one hundred and seventy-five (175) paraffin-embedded adjacent nontumor specimens in tissue chips (HLivH180Su10 and HLivH180Su06, respectively) were purchased from Shanghai Outdo Biotech (Shanghai, China). Clinical data were obtained from the medical records of the patients, and patients who had received preoperative radiation, chemotherapy, or biotherapy were excluded. All specimens had a confirmed pathological diagnosis. Data on some clinicopathological parameters, such as HCC recurrence, HBsAg positivity, recurrence status, AFP, total bilirubin, ALT, GGT, Edmondson-Steiner grade, tumor number and tumor size, were available in only approximately half of the HCC patients. Patient consent and ethics approval from the Ethics Committee of Shanghai Outdo Biotech Company were obtained.

### *In situ* hybridization (ISH)

*In situ* hybridization was conducted on paraffin-embedded specimens (4-μm thick) as previously described 19. Paraffinized sections were deparaffinized in xylene and rehydrated in a graded alcohol series and distilled water. After treatment with proteinase K at 37 °C for 30 min, the sections were rinsed, fixed and then prehybridized for 2 h. Hybridization was performed with miRCURY miR-5188 digoxygenin-labeled probes designed and synthesized by BersinBio (Guangzhou, China). Slides were then washed and incubated with anti-digoxygenin-HRP Fab fragments for 1 hour at room temperature. Signals were visualized by the addition of DAB substrate (Maixin Biotech. Co., Ltd., Fuzhou, China), and the staining intensity was scored as previously described 20. For statistical analysis, a score ≤ 6 indicated low expression, and a score > 6 indicated high expression.

### Immunohistochemistry (IHC) and evaluation of immunohistochemical staining

Paraffinized sample sections (4-μm thick) were deparaffinized and dehydrated, and antigen retrieval was then performed in citrate buffer for 3 min. Endogenous peroxidase activity and nonspecific antigens were blocked with 3% H_2_O_2_ and goat serum followed by incubation with antibodies (Table S3) overnight at 4 °C. After washing, the sections were incubated with HRP-conjugated secondary antibody and visualized using DAB substrate (Maixin Biotech. Co., Ltd., Fuzhou, China). The staining intensity was scored as previously described 20. For statistical analysis, a score ≤ 6 indicated low expression, and a score > 6 indicated high expression.

### Statistical analysis

All data were analyzed using SPSS 22.0 (SPSS, Inc., Chicago, IL, USA). The data are presented as the means±SDs. Statistical significance was detected using Student's two-tailed t-test for differences between two groups, one-way ANOVA for differences between multiple groups, the general linear model repeated measures variance analysis for differences in tumor growth and MTT assay results. Skewed data were analyzed by the Wilcoxon rank sum test. The kappa consistency test or Spearman's rank correlation test was used to examine correlations between gene expression. Correlations between gene expression and clinicopathological characteristics were assessed by the chi-square test. Log-rank tests of Kaplan-Meier survival curves were conducted to elucidate the relationship between gene expression and overall patient survival. Univariate and multivariate survival analyses were conducted using the Cox proportional hazards regression model. All statistical tests were two-sided, and a *P* value <0.05 indicated statistical significance (* *P*<0.05, ** *P*<0.01 and *** *P*<0.001).

## Results

### MiR-5188 is upregulated in HCC and confers poor patient prognosis

**Figure 1:**
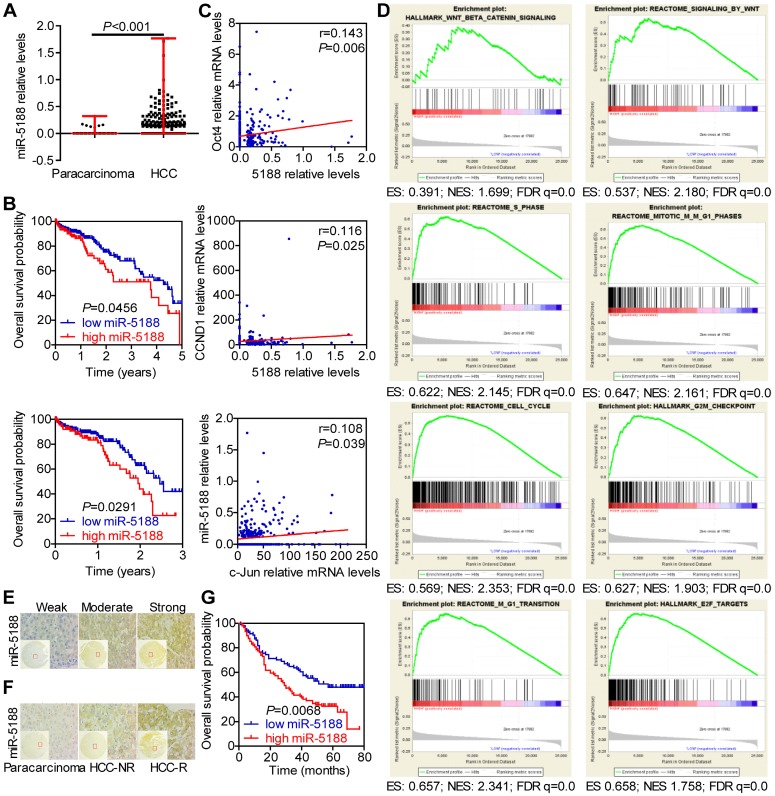
** MiR-5188 is upregulated in HCC and confers poor patient prognosis. (A)** Bioinformatics analysis of miR-5188 expression in HCC (n=374) and para-carcinoma tissues (n=50) (Wilcoxon rank sum test) based on data from TCGA database. **(B)** Kaplan-Meier survival analysis of HCC patient data from TCGA database based on miR-5188 expression (log-rank test).** (C)** The relationships among Oct4, Nanog, c-Jun and miR-5188 expression (Spearman's rank correlation test). **(D)** GSEA of miR-5188 shows significant enrichment of the gene set involving the regulation of Wnt/β-catenin signaling and the cell cycle. **(E)** Representative *in situ* hybridization images showing miR-5188 staining in HCC. **(F)** Representative images showing differences in miR-5188 expression between HCC and para-carcinoma tissues and recurrent HCC (HCC-R) and nonrecurrent HCC (HCC-NR). **(G)** Overall survival analysis of HCC patients based on miR-5188 expression by *in situ hybridization* (log-rank test) (n=185). Lines indicate median values, and whiskers indicate minimum and maximum values **(A)**.

To identify the role of miRNAs in HCC, we conducted TCGA data mining and found that miR-5188, which has not been reported on, is a potential oncomiR in HCC. MiR-5188 levels were elevated in HCC compared with those in para-carcinoma (Figure 1A). Moreover, 5-year and 3-year survival analyses suggested that miR-5188 is positively correlated with poor prognosis in HCC patients (Figure 1B). In addition, miR-5188 expression was found to be positively correlated with Oct4, CCND1 and c-Jun expression (Figure 1C). Gene set enrichment analysis (GSEA) further revealed that miR-5188 is positively involved in the regulation of Wnt/β-catenin signaling and the cell cycle (Figure 1D). We then detected miR-5188 expression in 185 HCC tumors (T) and para-carcinoma tissues (N) by *in situ* hybridization (Figure 1E-F). Consistent with findings in data from TCGA database, miR-5188 expression was upregulated in HCC tumors compared with that in para-carcinoma tissues (*P* < 0.001). MiR-5188 levels were higher in samples from patients with recurrent HCC (HCC-R) than those from patients with no demonstrable evidence of recurrence (HCC-NR) (*P* = 0.028) (Figure 1F). Moreover, QPCR revealed that miR-5188 is highly expressed in 9 HCC cell lines compared with its expression in the LO2 immortalized liver cell line, and elevated miR-5188 levels were detected in hepatitis B virus (HBV)-positive HCC cells (QGY7703, Hep3B, 97H, PLC/PRF/5, 97L and HCCLM3 cell lines), unlike HBV-negative HCC cells (HepG2, SMMC7721 and Huh7 cells) (Figure S1A).

Correlation analysis showed that miR-5188 expression is positively correlated with gender, American Joint Committee on Cancer (AJCC) stage, T classification, recurrence, and hepatitis B virus infection but not other parameters (Table S5). Survival analysis further indicated that HCC patients with high levels of miR-5188 expression exhibit shorter overall survival than those with low levels of miR-5188 expression (median survival of 29 months versus 57 months, respectively) (Figure 1G). More importantly, univariate and multivariate analyses revealed that miR-5188 expression acts as an independent prognostic factor for overall survival in HCC patients (Table S6).

### MiR-5188 promotes tumor stemness, metastasis, proliferation and chemoresistance

**Figure 2:**
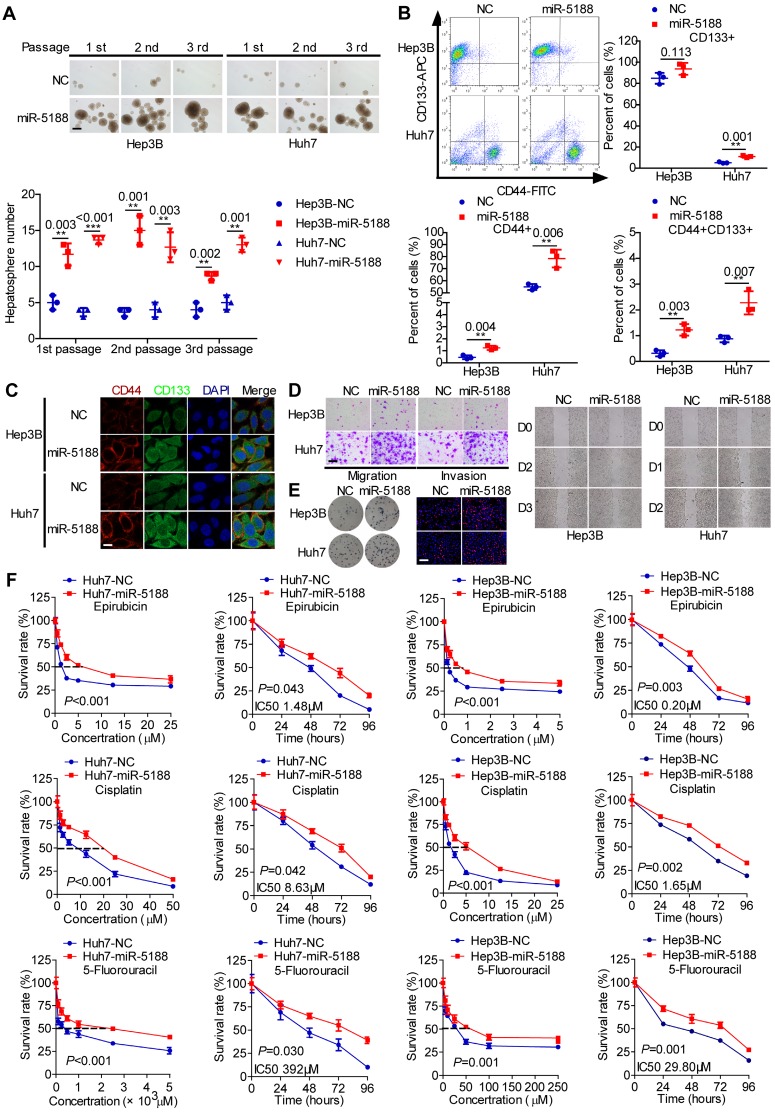
** MiR-5188 promotes stemness, metastasis, proliferation, and chemoresistance in HCC. (A-E): (A)** Hepatosphere formed during three serial passages (scale bar: 20 μm), **(B, C)** flow cytometry and immunofluorescence (scale bar: 5 μm) analyses, **(D)** Transwell assays (scale bar: 10 μm) and wound healing assays, and **(E)** colony formation assays and EdU incorporation assays (Scale bar: 10 μm) of miR-5188-overexpressing Hep3B and Huh7 cells and corresponding control cells. **(F)** Dose-dependent and time-dependent growth curves of Hep3B and Huh7 cells treated with epirubicin, cisplatin and 5-fluorouracil. Comparison of all groups vs. the control group by Student's t-test, n=3 independent experiments. All data are presented as the mean ± SD. Experiments were repeated three times.

To elucidate the function of miR-5188 in HCC, we introduced miR-5188 inhibitor, mimics or lentivirus particles into HCC cells and observed a greater than a two-fold change in miR-5188 expression in HCC cells transfected with miR-5188 mimics or the lentivirus-mediated delivery system (oe-miR-5188) compared with the corresponding control cells (Figure S1B-C). Upregulation of miR-5188 enhanced HCC stemness, metastasis, and proliferation (Figure 2A-E; Figure S1D-I) concomitant with an increase in resistance to the chemotherapy drugs 5-fluorouracil (5-FU), cisplatin (CDDP) and pharmorubicin (EPI) in a dose- and time-dependent manner (Figure 2F). However, miR-5188 knockdown in HCC cells induced the opposite effects (Figure S2).

**Figure 3:**
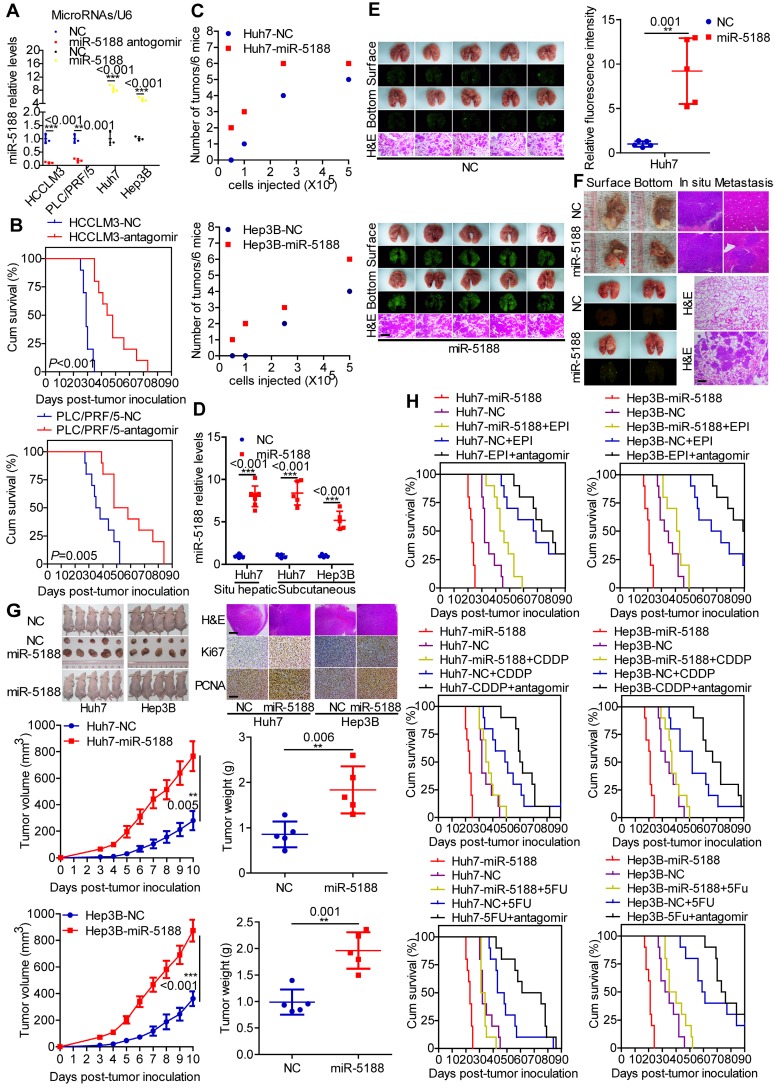
** MiR-5188 enhances the stemness, metastasis, proliferation, and chemoresistance of HCC cells *in vivo*. (A)** QPCR analysis of miR-5188 expression in xenograft tumors derived from HCCLM3 and PLC/PRF/5 cells treated with antagomir, Huh7 and Hep3B cells stably overexpressing miR-5188 and corresponding controls cells (n=3 independent experiments, Student's t-test). **(B)** Survival analysis showing the cumulative overall survival time of mice in the miR-5188 antagomir-treated group and the control group (n=10, log-rank test). **(C)** A subcutaneous xenograft mouse model was adopted to evaluate the effect of miR-5188 on tumor-initiating frequency (n=6). **(D)** QPCR analysis was used to confirm the miR-5188 expression level in tumors derived from miR-5188-overexpressing HCC cells and control cells. **(E)** A pulmonary metastasis model was adopted to evaluate the effect of miR-5188 on metastasis (n=5, Student's t-test). **(F)** An orthotopic tumor model was adopted to evaluate the effect of miR-5188 on proliferation and metastasis (n=7). **(G)** A subcutaneous xenograft mouse model was adopted to evaluate the effect of miR-5188 on proliferation (n=5, general linear model). Xenograft tumors were stained with H&E and underwent immunohistochemical analysis for Ki67 and PCNA expression (n=5). **(H)** Survival analysis indicating the cumulative overall survival time of mice in the oe-miR-5188 group, EPI/CDDP/5-FU-treated group, oe-miR-5188+EPI/CDDP/5-FU group, EPI/CDDP/5-FU combined with miR5188 antagomir-treated group and control group (n=10, log-rank test). All data are presented as the mean ± SD. Experiments were repeated three times **(A)** or conducted once **(B-H)**. H&E staining scale bars: 50 μm. Immunohistochemistry scale bars: 10 μm.

We then evaluated the *in vivo* oncogenic effect of miR-5188 in mouse models. QPCR verified decreased or increased miR-5188 expression in tumors derived from miR-5188 antagomir-treated or miR-5188-overexpressing HCC cells, respectively, compared with control tumors (Figure 3A). Specifically, depletion of miR-5188 by its antagomir prolonged the survival times of the mice (Figure 3B). In addition, miR-5188-overexpressing HCC cells presented an increased ability to form tumors in nude mice compared with that of the control cells (Figure 3C). Elevated miR-5188 expression levels were further confirmed in tumors derived from miR-5188-overexpressing HCC cells compared with miR-5188 expression in tumors obtained from control HCC cells (Figure 3D). The pulmonary metastasis model showed that in contrast to metastatic nodules in the control group, more metastatic nodules were detected in mice injected with oe-miR-5188-transfected Huh7 cells, which was confirmed by fluorescence and histopathologic findings (Figure 3E). Furthermore, mice injected with miR-5188-overexpressing HCC cells showed accelerated tumor growth, intrahepatic dissemination and extrahepatic metastasis compared with control cells in the established *in situ* hepatic cancer model. Seven out of seven (7/7) mice injected with oe-miR-5188-transfected Huh7 cells developed lung metastasis, whereas only four out of seven (4/7) mice in the control group developed lung metastasis (Figure 3F). In a subcutaneous xenograft mouse model, mice inoculated with oe-miR-5188-transfected HCC cells exhibited increased tumor burden and expression of Ki67 and PCNA compared with the control group (Figure 3G). Moreover, prolonged survival times were observed for mice in the EPI/CDDP/5-FU-treated group, with a shorter survival time observed in mice in the oe-miR-5188 group compared with the control mice. Intriguingly, combined chemotherapeutic treatment and miR-5188 antagomir administration delayed HCC progression. However, there was no significant difference between HCC progression in the oe-miR-5188+EPI/CDDP/5-FU group and the control group (Figure 3H).

### MiR-5188 augments β-catenin-mediated tumor stemness, metastasis, proliferation, chemoresistance and c-Jun signaling

**Figure 4:**
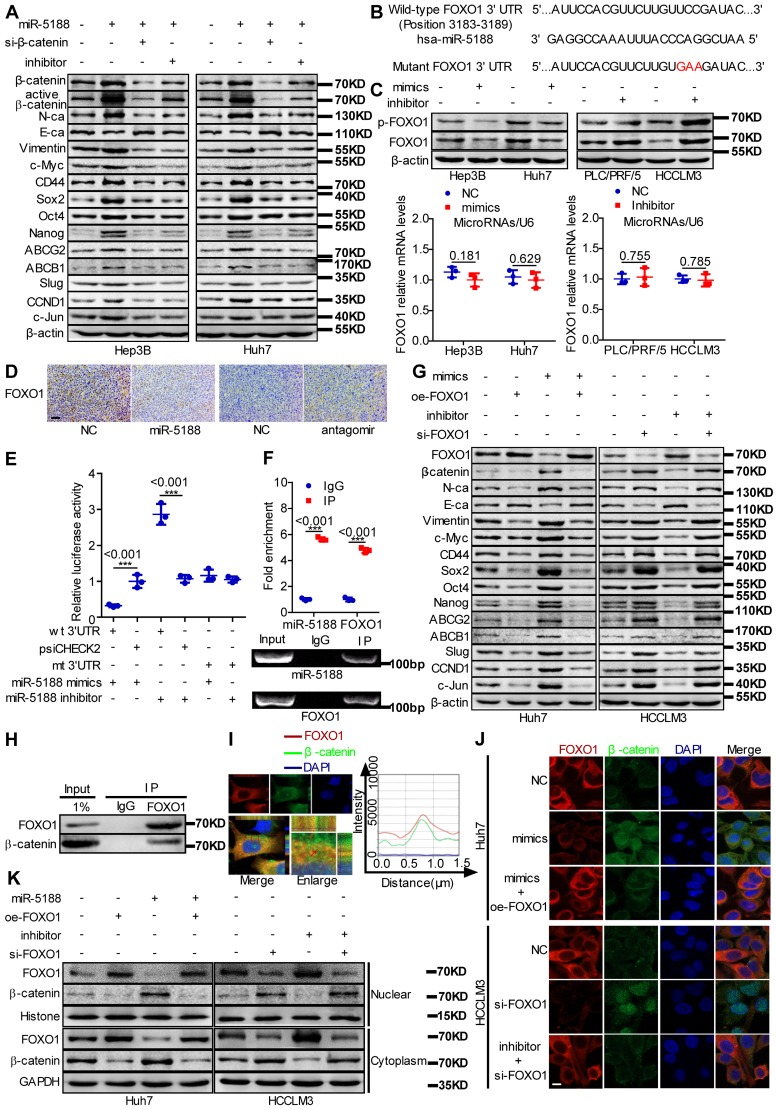
** MiR-5188 directly targets FOXO1 to augment β-catenin-mediated tumor stemness, metastasis, proliferation, and chemoresistance in HCC. (A)** Western blot analysis was utilized to examine stemness, metastasis, proliferation, chemoresistance, and Wnt/β-catenin signaling-associated protein expression levels in HCC cells. **(B)** Bioinformatics analysis was used to predict miR-5188-binding sequences within the FOXO1 3'-UTR. **(C)** QPCR and Western blot analysis were used to examine FOXO1 mRNA and protein levels in miR-5188-overexpressing HCC cells, miR-5188-silenced HCC cells, and the corresponding control cells (n=3 independent experiments, Student's t-test). **(D)** Immunohistochemical analysis detected FOXO1 protein expression in xenograft tumors derived from HCCLM3 cells treated with antagomir, Huh7 cells stably overexpressing miR-5188 and the corresponding control cells (scale bar: 10 μm) (n=5). **(E)** Luciferase reporter assays were conducted to validate the interaction between miR-5188 and the 3'UTR of FOXO1 (n=3 independent experiments, one-way ANOVA). **(F)** RIP was conducted to validate the interaction between AGO2-bound miR-5188 and FOXO1 mRNA (n=3 independent experiments, Student's t-test). **(G)** Western blot analysis of stemness, metastasis, proliferation, chemoresistance, and Wnt/β-catenin signaling-associated protein expression levels in HCC cells. **(H)** Endogenous coimmunoprecipitation was used to test the interaction between FOXO1 and β-catenin. **(I)** Immunofluorescence costaining was utilized to detect the colocalization of FOXO1 and β-catenin. Fluorescence intensities along the red arrow crossing the cytoplasm were calculated to show the colocalization of FOXO1 and β-catenin. **(J)** Immunofluorescence costaining was carried out to detect the expression and localization of FOXO1 and β-catenin (scale bar: 5 μm). **(K)** Nucleic and cytoplasmic β-catenin protein expression was detected by Western blotting. All data are presented as the mean ± SD. Experiments were repeated three times.

Western blotting data indicated that miR-5188 overexpression suppresses the expression of E-cadherin and induces the expression of β-catenin, N-cadherin, vimentin and the known Wnt targets c-Myc, CD44, Sox2, Oct4, Nanog, ABCG2, ABCB1, Slug, CCND1 and c-Jun. Simultaneous knockdown of β-catenin or introduction of miR-5188 inhibitor in oe-miR-5188-transfected HCC cells reversed changes in expression patterns mediated by miR-5188 upregulation (Figure 4A). Similarly, depletion of β-catenin or miR-5188 rescued miR-5188-induced stimulation of HCC stemness, metastasis, proliferation, and chemoresistance (Figure S3).

### MiR-5188 directly targets FOXO1 to induce β-catenin-mediated tumor stemness, metastasis, proliferation, chemoresistance and c-Jun signaling

FOXO1 was predicted to be a direct target of miR-5188 by RNAhybrid, TargetScan, Microt4 and miRmap (Figure 4B). MiR-5188 knockdown enhanced FOXO1 and p-FOXO1 protein levels, and miR-5188 overexpression induced the opposite effect in HCC cells; however, miR-5188 expression had no effect on FOXO1 mRNA expression (Figure 4C). Consistent with these *in vitro* findings, immunohistochemical analysis of xenografts originating from miR-5188-overexpressing or miR-5188-depleted HCC cells suggested a reduction or increase in FOXO1 expression, respectively (Figure 4D). FOXO1 was confirmed as a direct target of miR-5188 by the luciferase reporter assay (Figure 4E). RIP further confirmed the interaction between AGO2-bound miR-5188 and FOXO1 mRNA (Figure 4F).

We further found that FOXO1 can reverse the effect of miR-5188 on E-cadherin, β-catenin, N-cadherin, vimentin and known Wnt target expression (Figure 4G). Moreover, the TOP/FOP luciferase reporter assay confirmed the modulation of Wnt signaling by miR-5188 and FOXO1 in HCC cells (Figure S4A). Consistently, FOXO1 suppressed the miR-5188-induced stimulation of HCC stemness, migration and invasion, proliferation and chemoresistance (Figure S4B-E). These results suggest that FOXO1 overcomes the oncogenic effects of miR-5188 in HCC cells.

A previous report suggested that FOXO1 functionally interacts with β-catenin in oxidative stress signaling 21. We validated the interaction between FOXO1 and β-catenin via Co-IP (Figure 4H). Intriguingly, FOXO1 was abundant in the cytoplasm and colocalized with β-catenin in HCC cells (Figure 4I). Immunofluorescence triple staining and a cell fraction assay showed that FOXO1 restrains miR-5188-stimulated nuclear β-catenin enrichment (Figure 4J-K). Consistently, oe-miR-5188-transfected HCC cells exhibited higher levels of β-catenin, Sox2, Oct4, Nanog, CCND1, N-cadherin, vimentin, Ki67, and PCNA and lower levels of E-cadherin compared with those in control cells in the xenograft mouse model (Figure S4F).

### c-Jun directly induces miR-5188 expression and cooperatively drives β-catenin activation in HCC

**Figure 5:**
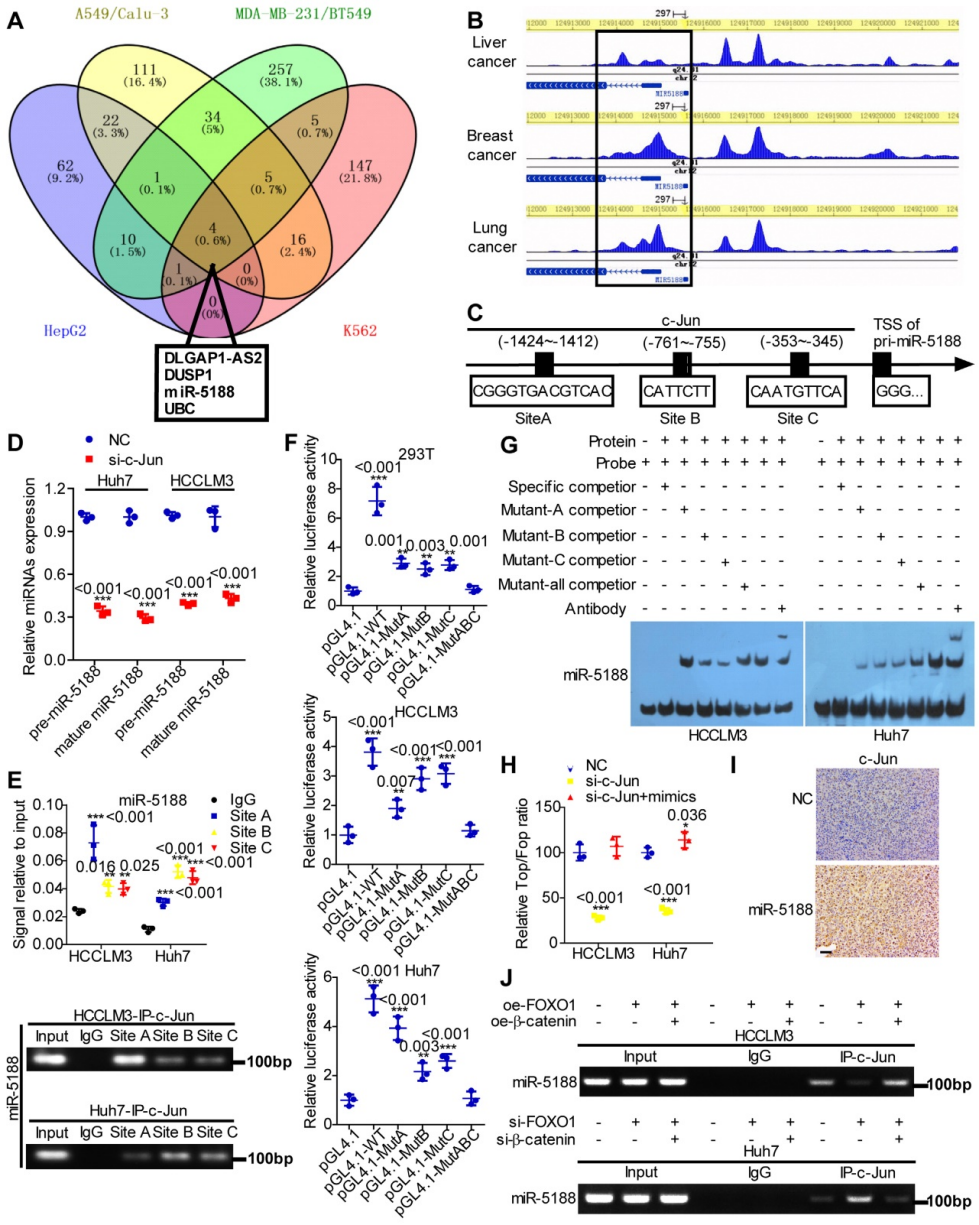
** c-Jun transcriptionally promotes miR-5188 expression to form a positive regulatory loop. (A)** Venn diagram analysis was used to screen c-Jun-regulated downstream effectors among the top putative targets based on the Cistrome Data Browser.** (B)** ChIP-seq binding peaks were searched by the Cistrome Data Browser. **(C)** Bioinformatics analysis was utilized to predict c-Jun-binding sites within the promoter of miR-5188. **(D)** QPCR was used to examine pre-miR-5188 and mature miR-5188 levels in c-Jun-silenced HCC cells and control cells (n=3 independent experiments, Student's t-test). **(E)** Chromatin immunoprecipitation (comparison of all groups vs. the IgG group) (n=3 independent experiments, one-way ANOVA) was used to identify c-Jun binding to the miR-5188 promoter. **(F)** Luciferase reporter assays (comparison of all groups vs. the control group) (n=3 independent experiments, one-way ANOVA) were performed to confirm c-Jun binding to the miR-5188 promoter. **(G)** Protein-DNA interactions between c-Jun and the miR-5188 promoter were determined using electrophoretic mobility shift assays. **(H)** TOP/FOP luciferase reporter assays were performed to detect Wnt/β-catenin signaling activity (n=3 independent experiments, one-way ANOVA). **(I)** Immunohistochemical analysis was used to detect c-Jun expression in xenograft tumors derived from Huh7 cells after stable miR-5188 overexpression and control cells (scale bar: 20 μm) (n=5). **(J)** Chromatin immunoprecipitation analysis assessed c-Jun binding to the miR-5188 promoter in FOXO1-overexpressing HCCLM3 cells, FOXO1-overexpressing HCCLM3 cells with β-catenin overexpression, FOXO1-silenced Huh7 cells, FOXO1-silenced Huh7 cells with β-catenin knockdown, and the corresponding control cells. All data are presented as the mean ± SD. Experiments were repeated three times.

Based on high-quality ChIP-seq data in the Cistrome Data Browser 22 (http://cistrome.org/db/), miR-5188 was among the top putative miRNA targets of c-Jun identified as having a promoter region harboring binding sites for c-Jun in HCC, lung cancer, breast cancer and erythroleukemia (Figure 5A). Moreover, the Cistrome Data Browser in conjunction with UCSC, PROMO, SnapGene and JASPAR bioinformatics software identified three c-Jun-binding sites inside the miR-5188 promoter region (Figure 5B-C). QPCR analysis showed that c-Jun knockdown decreases pre-miR-5188 and mature miR-5188 levels (Figure 5D). ChIP, luciferase reporter assays and EMSAs further confirmed that the c-Jun protein binds to all three sites in the miR-5188 promoter in HCCLM3 and Huh7 cells (Figure 5E-G). Taken together, these data suggest that c-Jun regulates miR-5188 transcription by functionally binding to the miR-5188 promoter in HCC.

We applied a TOP/FOP luciferase reporter assay to further delineate the specific role of c-Jun in Wnt signaling and found that miR-5188 restores the inhibitory effect of c-Jun knockdown on Wnt signaling (Figure 5H). Consistently, immunohistochemical analysis of xenografts originating from oe-miR-5188-transfected HCC cells suggested increased c-Jun expression (Figure 5I). Finally, ChIP revealed that β-catenin alleviates the inhibitory effect of FOXO1 on c-Jun binding to the miR-5188 promoter in HCC cells (Figure 5J). Overall, our data demonstrate that c-Jun and miR-5188 form a feedback loop to drive Wnt/β-catenin activation.

### HBX promotes tumor stemness, metastasis, proliferation, and chemoresistance through an miR-5188-modulated positive regulatory loop

**Figure 6:**
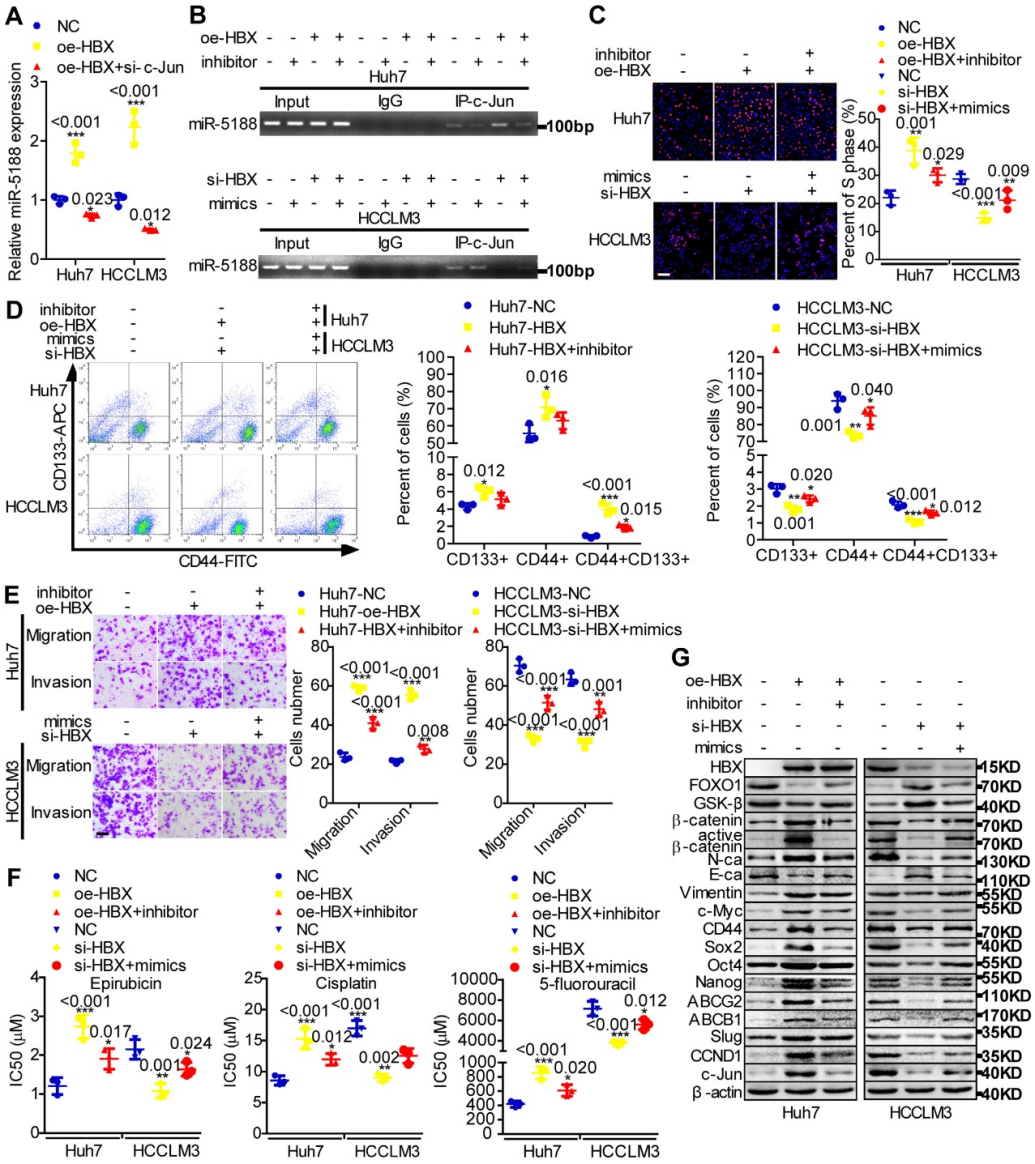
** The hepatitis B X protein augments miR-5188-mediated tumor stemness, metastasis, proliferation, chemoresistance and Wnt/β-catenin/c-Jun signaling in HCC. (A)** QPCR analysis examined miR-5188 expression in HBX-overexpressing HCC cells, HBX-overexpressing HCC cells with c-Jun knockdown and the corresponding control cells (one-way ANOVA).** (B)** Chromatin immunoprecipitation was used to validate c-Jun binding to the transcriptional regulatory region of miR-5188 in miR-5188-depleted Huh7 cells, HBX-overexpressing Huh7 cells, HBX-overexpressing Huh7 cells with miR-5188 depletion, miR-5188-overexpressing HCCLM3 cells, HBX-depleted HCCLM3 cells, HBX-depleted HCCLM3 cells with miR-5188 overexpression, and the corresponding control cells. EdU incorporation assays (scale bar: 10 μm) **(C),** flow cytometry analysis** (D)**, Transwell assays (scale bar: 10 μm) **(E)** and anticancer drug sensitivity tests **(F)** were used to detect stemness, migration, invasion and drug chemoresistance in HBX-overexpressing Huh7 cells, HBX-overexpressing Huh7 cells with miR-5188 knockdown, HBX-silenced HCCLM3 cells, HBX-silenced HCCLM3 cells with miR-5188 overexpression, and the corresponding control cells (one-way ANOVA). **(G)** Western blot analysis was performed to assess stemness, metastasis, proliferation, chemoresistance, and Wnt/β-catenin signaling-associated protein expression levels in HBX-overexpressing Huh7 cells, HBX-overexpressing Huh7 cells with miR-5188 knockdown, HBX-silenced HCCLM3 cells, HBX-silenced HCCLM3 cells with miR-5188 overexpression, and the corresponding control cells. Comparison of all groups vs. the control group by one-way ANOVA, n=3 independent experiments. All data are presented as the mean ± SD. Experiments were repeated three times.

MiR-5188 expression was shown to be positively correlated with hepatitis B virus infection (Table S5). HBx, a key HBV-encoded oncogenic protein, is essential for Wnt signaling activation 10. Therefore, we wondered whether HBx and the miR-5188-FOXO1/β-catenin-c-Jun feedback loop are connected. We then found that c-Jun knockdown impairs HBX-induced stimulation of miR-5188 levels (Figure 6A) and that miR-5188 can regulate the HBX-induced increase in c-Jun-mediated transcription of miR5188 in HCC cells (Figure 6B). In addition, miR-5188 mediated the oncogenic effects of HBX on HCC stemness, metastasis, proliferation, chemoresistance, and Wnt/β-catenin and c-Jun signaling (Figure 6C-G). Moreover, expression of the miR-5188-targeting protein FOXO1 was attenuated in HBX-overexpressing HCC cells and increased in HBX-suppressed HCC cells (Figure 6G), demonstrating that HBX modulates the miR-5188/FOXO1/β-catenin/c-Jun feedback loop to drive Wnt/β-catenin activation.

### Correlations between miR-5188, FOXO1, and β-catenin expression and clinicopathological characteristics in HCC

**Figure 7:**
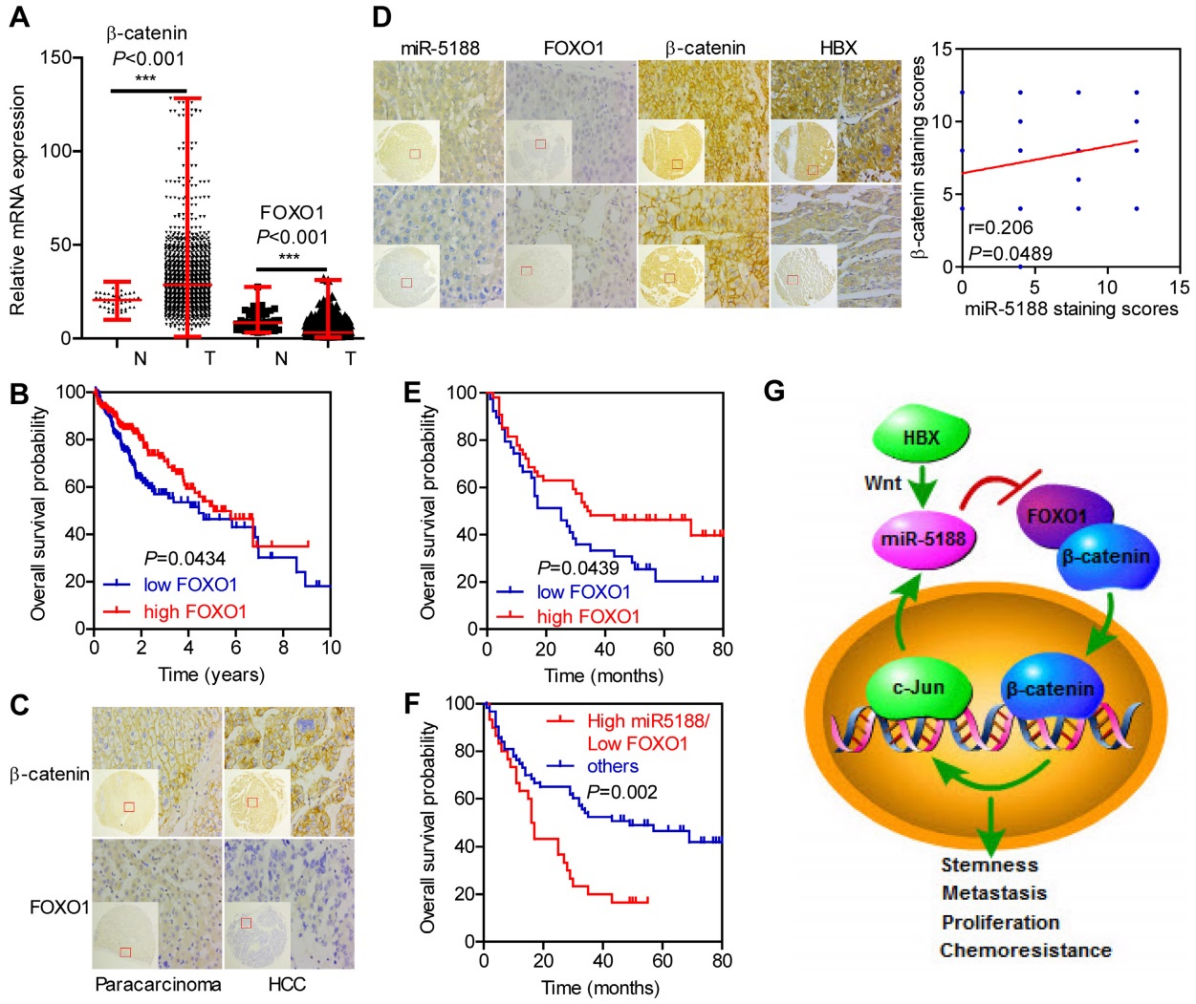
** Immunohistochemical staining for FOXO1, β-catenin and HBX expression and *in situ hybridization* analysis of miR-5188. (A)** Comparison of β-catenin and FOXO1 expression between HCC and para-carcinoma tissues from data in TCGA database (Wilcoxon rank sum test). **(B)** Kaplan-Meier survival analysis of HCC patients based on FOXO1 expression from data in TCGA database (log-rank test). **(C)** Representative immunohistochemical images of β-catenin and FOXO1 expression between HCC tissues and para-carcinoma tissues. **(D)** Correlations among FOXO1, β-catenin, HBX and miR-5188 expression (representative *in situ hybridization* images) (Spearman's rank correlation test). **(E)** Kaplan-Meier survival analysis of HCC patients based on FOXO1 expression (log-rank test). **(F)** Kaplan-Meier survival analysis of HCC patients based on miR-5188 and FOXO1 expression (log-rank test). **(G)** Working model of miR-5188-FOXO1/β-catenin-c-Jun feedback loop induced by the hepatitis B X protein through Wnt signaling in hepatocellular carcinoma. Lines indicate median values, and whiskers indicate minimum and maximum values **(A)**.

Based on analysis of data from TCGA database, β-catenin levels were elevated, but FOXO1 levels were downregulated in HCC (T) compared with those in para-carcinoma (N) (Figure 7A). Moreover, FOXO1 expression was negatively correlated with poor prognosis in HCC patients (Figure 7B).

We then performed ISH and IHC in 185 HCC tumors and 175 para-carcinoma tissues. Consistently, β-catenin levels were upregulated (*P* < 0.001), but FOXO1 levels were downregulated in HCC compared with those in para-carcinoma tissues (*P* < 0.001) (Figure 7C). Moreover, miR-5188 expression was negatively correlated with FOXO1 expression (*kappa* = -0.229, *P* = 0.028) and positively correlated with HBX (*kappa* = 0.201, *P* = 0.047) and the nuclear translocation of β-catenin (*kappa* = 0.215, *P* = 0.040). FOXO1 expression was negatively correlated with β-catenin expression (*kappa* = -0.278, *P* = 0.007) (Figure 7D).

Survival analysis further indicated that high FOXO1 expression levels are negatively correlated with poor prognosis in HCC patients (median survival of 35 months versus 25 months for controls versus HCC patients, respectively) (Figure 7E). Next, we divided the patients into two groups; one group was composed of patients with high miR-5188 and low FOXO1 expression levels and named High miR5188/Low FOXO1, and a second group was composed of the remaining patients and named 'others'. Survival analysis indicated that high miR-5188 and low FOXO1 expression levels contribute to a shorter overall survival time in HCC patients (median survival of 16.5 months versus 49 months in the High miR5188/Low FOXO1 group versus others, respectively) (Figure 7F).

## Discussion

In this study, we first verified that miR-5188 is significantly upregulated in HCC and that its expression is associated with poor prognosis in HCC patients. MiR-5188 augmented Wnt/β-catenin and its downstream signals, tumor stemness, EMT, and c-Jun, which facilitated HCC progression. Specifically, inhibition of miR-5188 restrained HCC development, suggesting that miR-5188 can be used as a therapeutic target in HCC.

FOXO1 is a well-characterized tumor suppressor that suppresses tumor stemness, metastasis, proliferation and chemoresistance in various cancers 23-27 including HCC 28, 29. However, the role of FOXO1 in the regulation of HCC stemness has not been reported. Here, we demonstrate that FOXO1, which is directly targeted by miR-5188, suppresses Wnt/β-catenin and its downstream signals, tumor stemness, EMT and c-Jun, which reduced the CSC properties, metastasis, growth and chemoresistance of HCC. One of the earliest reports on FOXO1 suggested that FOXO1 functionally interacts with β-catenin in oxidative stress signaling 21. Several subsequent studies indicated that FOXO1 couples with β-catenin to raise the transcriptional activity of FOXO1 and repress the transcriptional activity of transcription factors including T cell factor/lymphoid enhancer binding factor. Surprisingly, elevated FOXO1 does not affect the nuclear accumulation of β-catenin in cells 30, 31. Inconsistent with the results of previous studies, FOXO1 downregulated β-catenin expression in this investigation. Furthermore, FOXO1 interacted with β-catenin in the cytoplasm to impair the nuclear accumulation of β-catenin, thus downregulating Wnt signaling activity, especially downstream c-Jun signaling, and proteins associated with stemness, metastasis, proliferation, and chemoresistance.

c-Jun contributes to liver tumor development 32, 33 and functions as an essential transcription factor that modulates many protein-coding genes as well as the expression of miRNAs to regulate HCC stemness 34, metastasis 35, proliferation 36, and chemoresistance 37. In prior work, we identified miR-5188 as a potential oncomiR with a promoter region harboring multiple putative binding sites for the pro-oncogene c-Jun based on the Cistrome Data Browser. Subsequent experiments verified that c-Jun positively regulates the biogenesis of miR-5188 by directly binding to its promoter region. These findings demonstrate that miR-5188 directly targets FOXO1 and couples with the FOXO1/β-catenin complex and transcription factor c-Jun to form a positive regulatory loop. This loop augments Wnt/β-catenin and c-Jun signaling activity to facilitate the CSC properties, metastasis, growth, and chemoresistance of HCC cells.

Wnt/β-catenin signaling is frequently hyperactivated in HCC CSCs 38. Several studies have suggested a connection between HBX, miRNAs and HCC progression 39, 40. In the present work, we show that miR-5188 is regulated by c-Jun, a downstream effector of the Wnt/β-catenin signaling pathway 41 and identify FOXO1 as the direct target of miR-5188. Moreover, GSEA suggested that miR-5188 is positively involved in the regulation of Wnt/β-catenin signaling, and correlation analysis showed that miR-5188 expression is positively correlated with hepatitis B virus infection. In addition, HBX was shown to promote miR-5188 expression to downregulate FOXO1 expression. Collectively, these results demonstrate that HBX promotes activation of Wnt/β-catenin and c-Jun signaling activity through the miR-5188/FOXO1/β-catenin/c-Jun feedback loop, facilitating HCC progression.

## Conclusions

In summary, our study elucidates the powerful function of miR-5188 in driving HCC stemness and progression. MiR-5188 antagomir specifically targeted miR-5188, markedly prolonged the survival time of HCC-bearing mice and improved HCC cell chemosensitivity *in vivo*. Moreover, we present a novel mechanism by which HBX-stimulated β-catenin-c-Jun signaling induces an miR-5188-FOXO1/β-catenin-c-Jun feedback loop that collaboratively promotes HCC progression by allowing the autonomous activation of Wnt/β-catenin signaling (Figure 7G). More importantly, transcriptomics data and our clinical data supported the role of miR-5188 as an oncomiR promoting HBV-modulated HCC progression that serves as an unfavorable prognostic factor for overall survival in HCC patients. Our discovery contributes to understanding the underlying role of specific miRNAs and proteins in regulatory networks and highlights the clinical and biological bases for the potential use of miR-5188 as a novel diagnostic factor and useful therapeutic target for HCC.

## Abbreviations

CSCs: cancer stem cells
ChIP: chromatin immunoprecipitation
Co-IP: coimmunoprecipitation
CDDP: cisplatin
EMSA: electrophoretic mobility shift assay
EPI: pharmorubicin
GSEA: gene set enrichment analysis
HCC: hepatocellular carcinoma
HBX: hepatitis X protein
HBV: hepatitis B virus
ISH: *in situ* hybridization
IHC: immunohistochemistry
RIP: RNA immunoprecipitation
5-FU: 5-fluorouracil
