# Supplementary material for: HBX-induced miR-5188 impairs FOXO1 to stimulate β-catenin nuclear translocation and promotes tumor stemness in hepatocellular carcinoma
Source: Theranostics. 2019 Oct 12;9(25):7583–98. doi: 10.7150/thno.37717 (PMC6831466; doi:10.7150/thno.37717)
Supplement: Supplementary file 1 — Supplementary figures and tables. [file thnov09p7583s1.pdf]

Supplementary figures

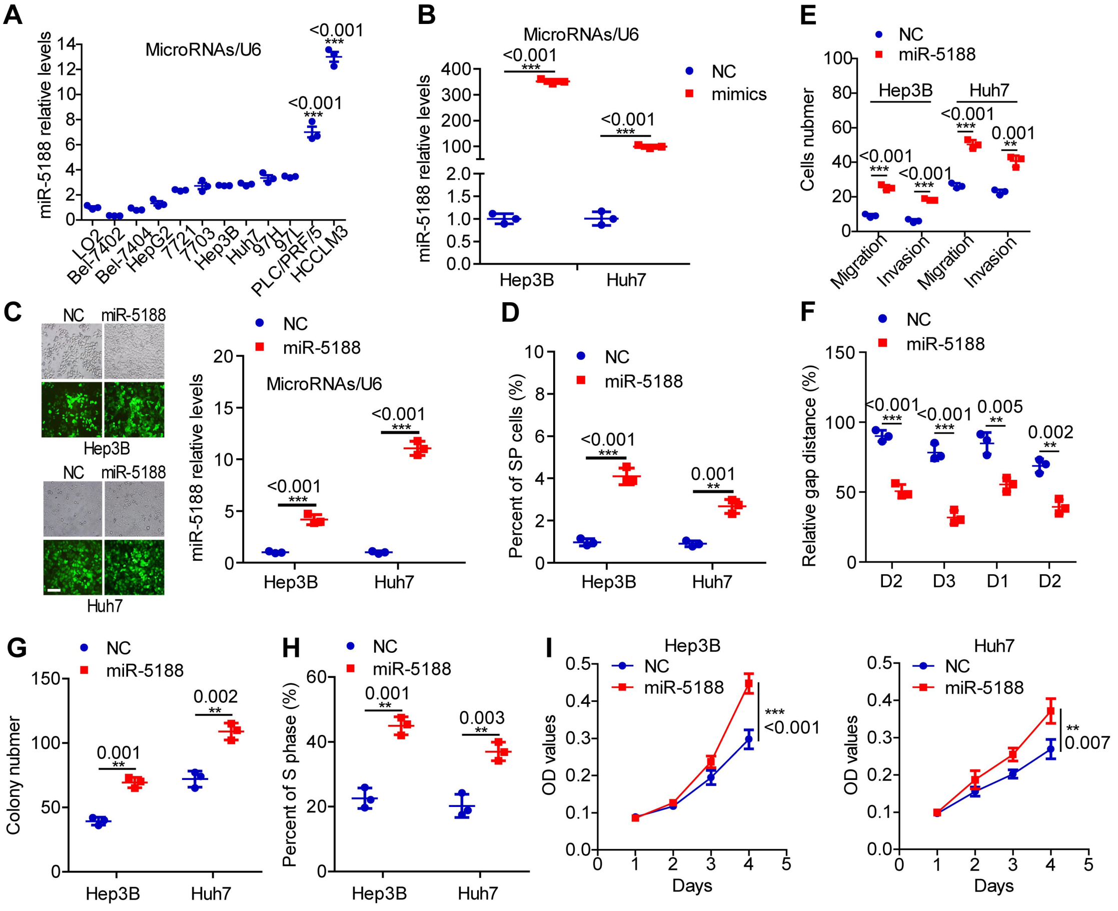

Figure S1. MiR-5188 promotes stemness, metastasis, proliferation, and chemoresistance in HCC. (A) QPCR analysis confirmed miR-5188 expression in Bel-7402, Bel-7404, HepG2, SMMC-7721, QGY-7703, Hep3B, Huh7, MHCC97H, PLC/PRF/5, MHCC97L, HCCLM3 and LO2 cells. **(B)** MiR-5188 expression in HCC cells treated with miR-5188 mimics and control cells. **(C)** MiR-5188 expression was examined in HCC cells after stable miR-5188 overexpression and control cells (scale bar: 20 μm). **(D-H)** Statistical analysis of the percentage of cells in side populations **(D)** and Transwell **(E)**, wound healing **(F)**, colony formation **(G)** and EdU incorporation assays **(H)** were performed in miR-5188-overexpressing Hep3B and Huh7 cells and the corresponding control cells. **(I)** MTT assays of miR-5188-overexpressing Hep3B and Huh7 cells and the corresponding control cells (n=3 independent experiments, general linear model). Comparison of all groups vs. the control group by Student's t-test, n=3 independent experiments. All data are presented as the mean ± SD. Experiments were repeated three times.

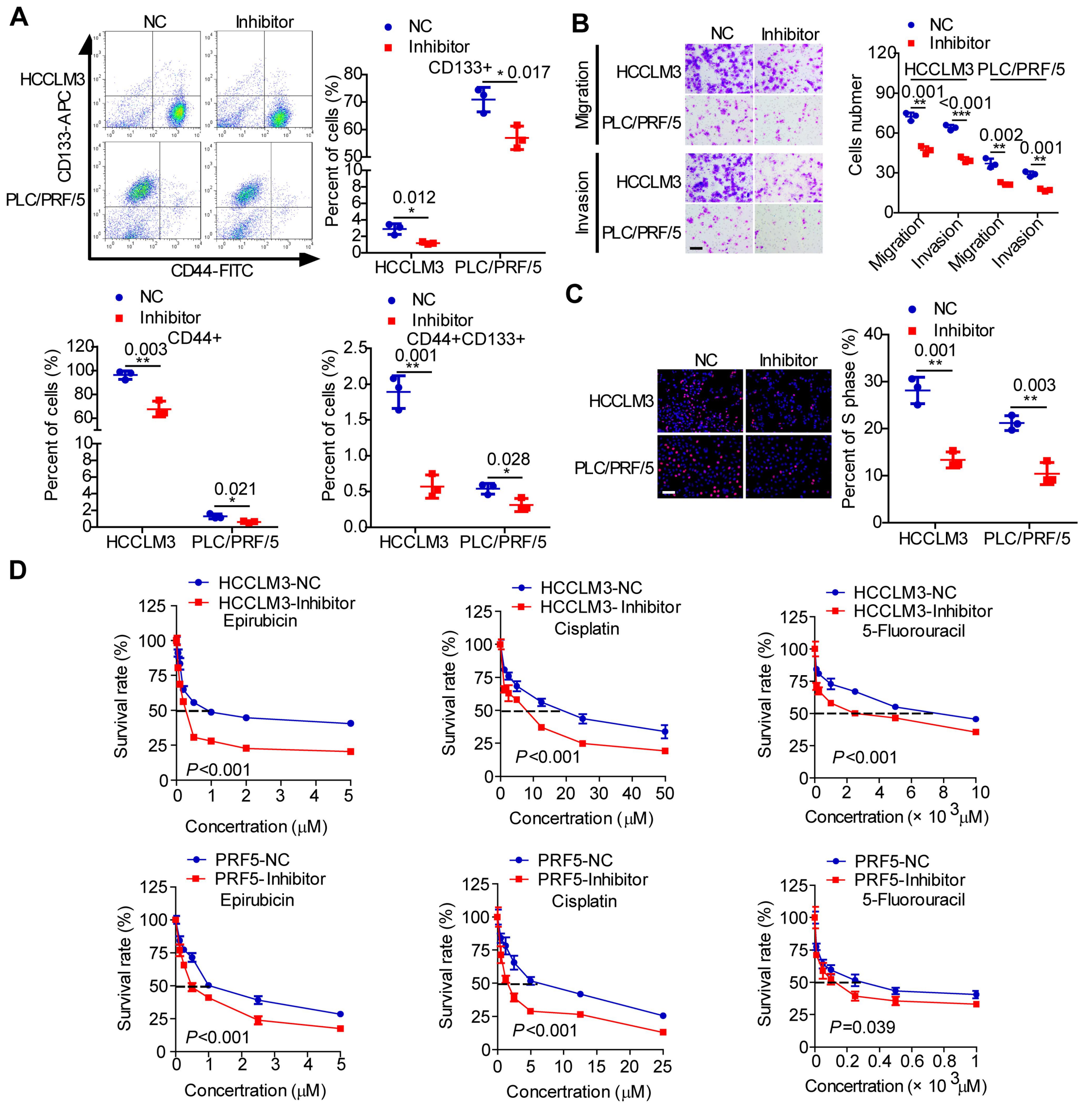

Figure S2. MiR-5188 knockdown inhibits stemness, metastasis, proliferation, and chemoresistance in HCC cells. Flow cytometry **(A)**, Transwell assays (scale bar: 10 μm) **(B)**, EdU incorporation assays (scale bar: 10 μm) **(C)** and anticancer drug sensitivity tests **(D)** were performed in miR-5188-silenced Hep3B and Huh7 cells and the corresponding control cells. Comparison of all groups vs. the control group by Student's t-test, n=3 independent experiments. All data are presented as the mean ± SD. Experiments were repeated three times.

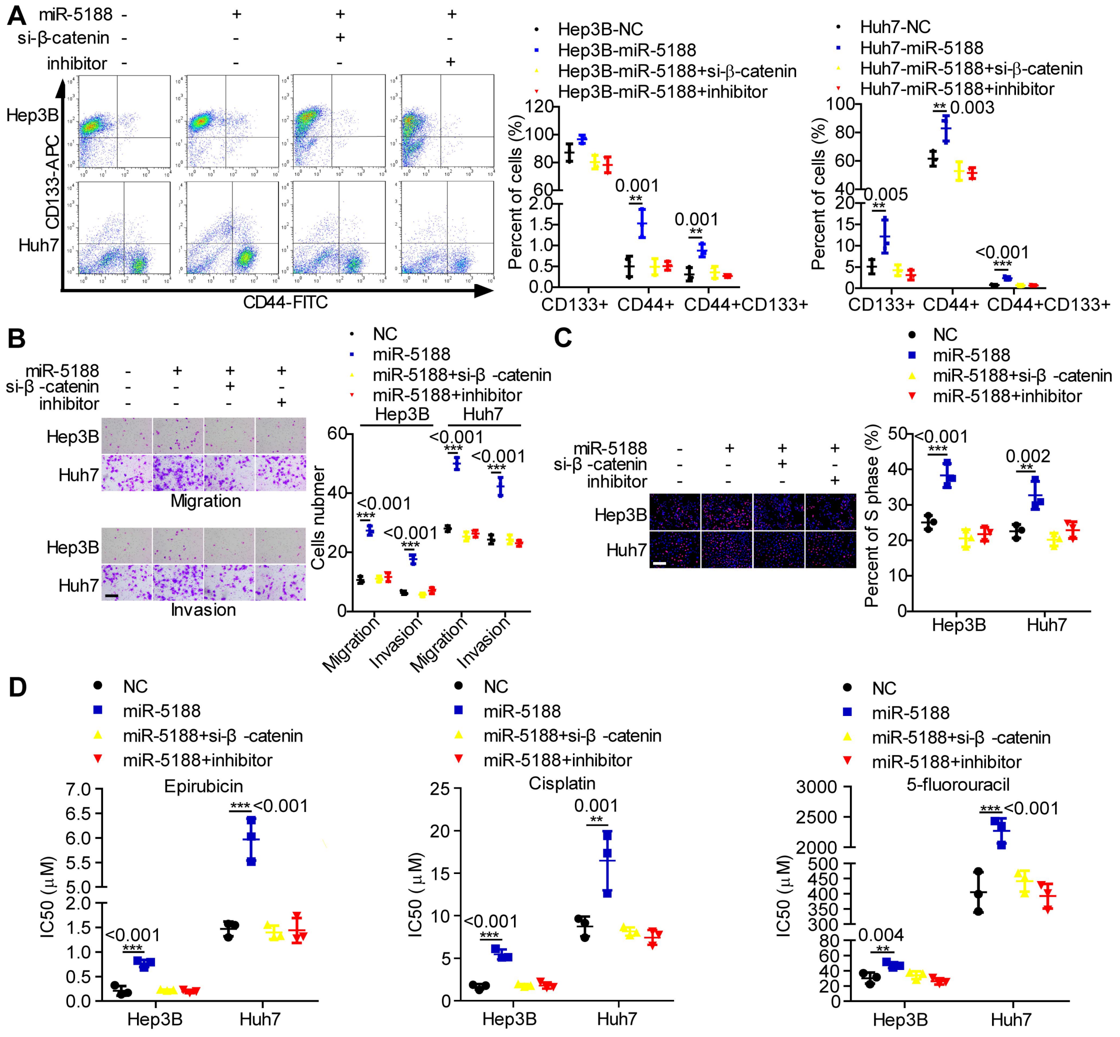

Figure S3. MiR-5188 accelerates β-catenin-mediated tumor stemness, metastasis, proliferation, chemoresistance and Wnt/β-catenin signaling in HCC. Flow cytometry analysis **(A)**, Transwell assays (scale bar: 10 μm) **(B)**, EdU incorporation assays (scale bar: 10 μm) **(C)** and anticancer drug sensitivity tests **(D)** were carried out in miR-5188-overexpressing HCC cells, miR-5188-overexpressing HCC cells with β-catenin knockdown, miR-5188-overexpressing HCC cells with miR-5188 knockdown, and the corresponding control cells (n=3 independent experiments, one-way ANOVA). All data are presented as the mean ± SD. Experiments were repeated three times.

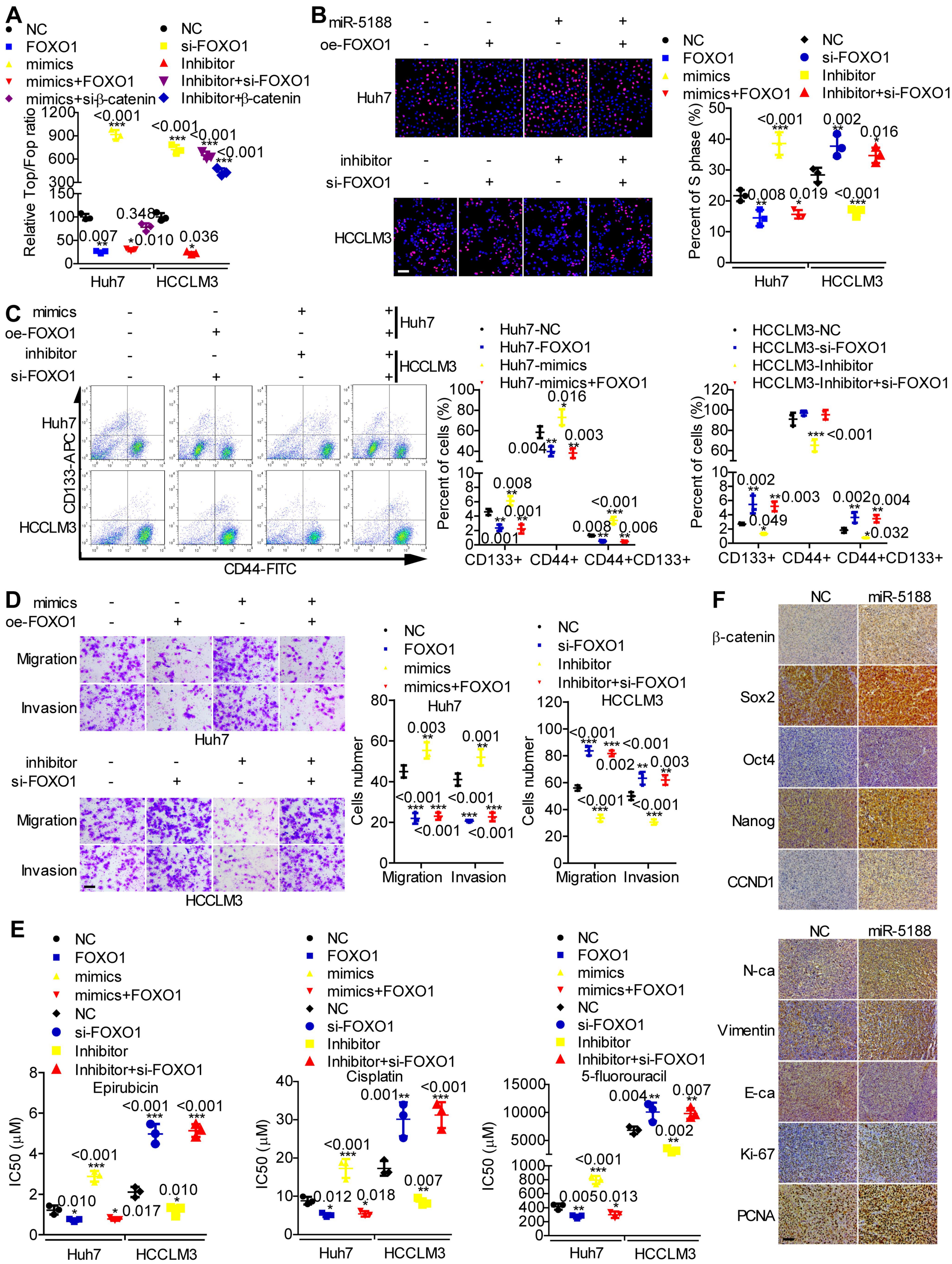

Figure S4. MiR-5188 regulates FOXO1-mediated tumor stemness, metastasis, proliferation, chemoresistance and Wnt/β-catenin signaling in HCC. TOP/FOP luciferase reporter assay showing Wnt/β-catenin signaling activity **(A)**, EdU incorporation assays (scale bar: 10 μm) **(B),** flow cytometry analysis **(C)**, Transwell assays (scale bar: 10 μm) **(D)** and anticancer drug sensitivity tests **(E)** were performed in FOXO1-overexpressing Huh7 cells, miR-5188-overexpressing Huh7 cells, miR-5188-overexpressing Huh7 cells with FOXO1 overexpression, FOXO1-silenced HCCLM3 cells, miR-5188-silenced HCCLM3 cells, miR-5188-silenced HCCLM3

cells with FOXO1 knockdown, and the corresponding control cells (n=3 independent experiments, one-way ANOVA) (comparison of all groups vs. the NC group). **(F)** Immunohistochemical analysis was used to examine β-catenin, Sox2, Oct4, Nanog, CCND1, E-ca, N-ca, vimentin, Ki67 and PCNA protein expression levels in xenograft tumors derived from Huh7 cells stably overexpressing miR-5188 and control cells (scale bar: 10 μm) (n=5). All data are presented as the mean ± SD. Experiments were repeated three times.

Supplementary Tables

Table S1. The sequences used in this study.

c-Jun	1	Sense	5’ GGCACAGCUUAAACAGAAA dTdT 3’
		Antisense	3’ dTdT CCGUGUCGAAUUUGUCUUU 5’
	2	Sense	5’ CGCAGCAGUUGCAAACAUU dTdT 3’
		Antisense	3’ dTdT GCGUCGUCAACGUUUGUAA 5’
FOXO1	1	Sense	5’CUGCAUCCAUGGACAACAA dTdT 3’
		Antisense	3’ dTdTGACGUAGGUACCUGUUGUU 5’
	2	Sense	5’ CCAGAUGCCUAUACAAACA dTdT 3’
		Antisense	3’ dTdT GGUCUACGGAUAUGUUUGU 5’
HBX	1	Sense	5’ GCACUUCGCUUCACCUCUG dTdT 3’
		Antisense	3’ dTdT CGUGAAGCAAGUGGAGAC 5’
	2	Sense	5’ -GGUCUUACAUAAGAGGACU dTdT 3’
		Antisense	3’ dTdT CCAGAAUGUAUUCUCCUGA 5’
β-catenin	1	Sense	5’ GAUGGUGUCUGCUAUUGUA dTdT 3’
		Antisense	3’ dTdT CUACCACAGACGAUAACAU 5’
	2	Sense	5’ GGACAAGGAAGCUGCAGAA dTdT 3’
		Antisense	3’ dTdT CCUGUUCCUUCGACGUCUU 5’
miR-5188 mimics		Sense	5‘AAUCGGACCCAUUUAAACCGGAG 3’
		Antisense	3’UUAGCCUGGGUAAAUUUGGCCUC 5’
Negative control		Sense	5’ UUUGUACUACACAAAAGUACUG 3’
		Antisense	3’ AAACAUGAUGUGUUUUCAUGAC 5’
miR-5188 inhibitor			5’ CUCCGGUUUAAAUGGGUCCGAUU 3’
Inhibitor negative control			5’ CAGUACUUUUGUGUAGUACAAA 3’
miR-5188 precursor			5’GGGAGGCAUGGAAAUUUCUCUGGUUUCAA UGGGUACGAUUAUUGUAAGCAGGAUCCAUU CAAUAAUCGGACCCAUUUAAACCGGAGAUU UUAAAAGACAGGAAUAGAAUCCCA 3’

Table S2. The primers used in this study.

Primers name		Sequence (5’-3’)
c-Jun	Forward	TCAGACAGTGCCCGAGATG
	Reverse	CTGCTGCGTTAGCATGAGTT
FOXO1	Forward	TGAACCGCCTGACCCAA
	Reverse	CAATGAACATGCCATCCAAG
HBX	Forward	CACTTCGCTTCACCTCTGC
	Reverse	TCGGTCGTTGACATTGCTG
β-catenin	Forward	GGCCCAGAATGCAGTTCGCCTT
	Reverse	AATGGCACCCTGCTCACGCA
β actin	Forward	CTCGCTGTCCACCTTCCA
	Reverse	ACCTTCACCGTTCCAGTTTT
miR-5188		AATCGGACCCATTTAAACCGGAG
U6	Forward	CTCGCTTCGGCAGCACA
	Reverse	AACGCTTCACGAATTTGCGT
Pre-miR-5188	Forward	TCTGGTTTCAATGGGTACG
	Reverse	TCTCCGGTTTAAATGGGTC
promoter of miR-5188-A	Forward	TGCGACGGAGAAAAGCC
	Reverse	GGGACCCTGACGTGAAGTT
promoter of miR-5188-B	Forward	GAGTCACCCAAGTCCCGTCCTA
	Reverse	AGCGAGCGTCCTGATCCTTC
promoter of miR-5188-C	Forward	TGCGAGATGGACGGGTCTT
	Reverse	AGGCTCAGGGAGGTTGAAGG

Table S3. A list of antibodies used for WB, ChIP, EMSA, Co-IP, ICC and IHC.

Antibodies name	Cat. No	Company	Species	Dulution
Flag	F7425	Sigma	Rabbit	1:1000 (WB); 1:20 (Co-IP)
HBX	sc-57760	Santa Cruz	Mouse	1:1000 (WB); 1:20 (Co-IP); 1:100 (ICC)
Ki67	ab16667	Abcam	Rabbit	1:100 (IHC)
c-Jun	9165	CST	Rabbit	1:1000 (WB); 1:50 (ChIP); 1:15 (EMSA); 1:20 (Co-IP); 1:300 (IHC)
β-catenin	8480	CST	Rabbit	1:1000 (WB); 1:100 (ICC); 1:100 (IHC)
β-catenin	2677	CST	Mouse	1:200 (ICC)
Active β-catenin	19807	CST	Rabbit	1:1000 (WB)
CD44	3570	CST	Mouse	1:1000 (WB)
ABCG2	42078	CST	Rabbit	1:1000 (WB)
FOXO1	2880	CST	Rabbit	1:1000 (WB); 1:20 (Co-IP); 1:100 (ICC); 1:100 (IHC)
Slug	9585	CST	Rabbit	1:1000 (WB)
PCNA	13110	CST	Rabbit	1:1000 (IHC)
c-Myc	10828-1-AP	Proteintech	Rabbit	1:1000 (WB)
CCND1	60186-1-Ig	Proteintech	Mouse	1:1000 (WB)
CCND1	ab134175	Abcam	Rabbit	1:100 (IHC)
SOX2	20118-1-AP	Proteintech	Rabbit	1:1000 (WB); 1:100 (IHC)
OCT4	11263-1-AP	Proteintech	Rabbit	1:1000 (WB); 1:100 (IHC)
Nanog	14295-1-AP	Proteintech	Rabbit	1:1000 (WB); 1:100 (IHC)
ABCB1	22336-1-AP	Proteintech	Rabbit	1:1000 (WB)
E-cadherin	60335-1-Ig	Proteintech	Mouse	1:1000 (WB); 1:100 (IHC)
N-cadherin	66219-1-Ig	Proteintech	Mouse	1:1000 (WB); 1:100 (IHC)
Vimentin	10366-1-AP	Proteintech	Rabbit	1:1000 (WB); 1:1000 (IHC)
CD133	18470-1-AP	Proteintech	Rabbit	1:1000 (WB)
GAPDH	60004-1-Ig	Proteintech	Mouse	1:5000 (WB)
Histone	17168-1-AP	Proteintech	Rabbit	1:1000 (WB)
β-actin	60008-1-Ig	Proteintech	Mouse	1:5000 (WB)

ChIP: chromatin immunoprecipitation
EMSA: electrophoretic mobility shift assay
ICC: immunofluorescence
IHC: immunohistochemistry
Co-IP: co-immunoprecipitation
WB: western blot

Table S4. The sequences used in Electrophoretic mobility shift assay (5'-3').

miR-5188	probes	wild type	ACGGGTGACGTCACGACAGCCCTAGAGTCACCCAAATTTTTGGGTCACATTT
	competitors	wild type	ACGGGTGACGTCACGACAGCCCTAGAGTCACCCAAATTTTTGGGTCACATTT
		site 1 mutant	ACGGGCTGTTCTTCGACAGCCCTAGAGTCACCCAAATTTTTGGGTCACATTT
		site 2 mutant	ACGGGTGACGTCACGACAGCCCTCATTCTTCCCAAATTTTTGGGTCACATTT
		site 3 mutant	ACGGGTGACGTCACGACAGCCCTAGAGTCACCCAAATTTTCAATGTTCATTT
		all sites mutant	ACGGGCTGTTCTTCGACAGCCCTCATTCTTCCCAAATTTTCAATGTTCATTT
MYH9	probes	wild type	AGGGCAGCGTCTGTGACTGACTCATTATGAGCTCACATAATCATCATGACAT CATCATAGCAAATACCTAGATGGACAGTGTTGTGAATCAGGCACGGGAAC
	competitors	wild type	AGGGCAGCGTCTGTGACTGACTCATTATGAGCTCACATAATCATCATGACAT CATCATAGCAAATACCTAGATGGACAGTGTTGTGAATCAGGCACGGGAAC
		site 1 mutant	AGGGCAGCGTAGAGAGTCCTCTAGGTATGAGCTCACATAATCATCATGACAT CATCATAGCAAATACCTAGATGGACAGTGTTGTGAATCAGGCACGGGAAC
		site 2 mutant	AGGGCAGCGTCTGTGACTGACTCATTATGAGCTCACATAATCATCCCACACG TCGTCCAGCAAATACCTAGATGGACAGTGTTGTGAATCAGGCACGGGAAC
		site 3 mutant	AGGGCAGCGTCTGTGACTGACTCATTATGAGCTCACATAATCATCATGACAT CATCATAGCAAATACCTAGATGGACAAGAGGACACCGAGAGCACGGGAAC
		all sites mutant	AGGGCAGCGTAGAGAGTCCTCTAGGTATGAGCTCACATAATCATCCCACACG TCGTCCAGCAAATACCTAGATGGACAAGAGGACACCGAGAGCACGGGAAC
Ubiquitin	probes	wild type	GGGACTTGGGTGACTCTAGGGCACTGGTATGTCACAAAGCTGGTGACTCAGC CTTTAAACACTGAAAACGGATCCAGTGACTCATCCCGATTCTTGCACA
	competitors	wild type	GGGACTTGGGTGACTCTAGGGCACTGGTATGTCACAAAGCTGGTGACTCAGC CTTTAAACACTGAAAACGGATCCAGTGACTCATCCCGATTCTTGCACA
		site 1 mutant	GGGACTTGGGGCGGAGAAGGGCACTGGTATGTCACAAAGCTGGTGACTCAG CCTTTAAACACTGAAAACGGATCCAGTGACTCATCCCGATTCTTGCACA
		site 2 mutant	GGGACTTGGGTGACTCTAGGGCACTGGTATGTCACAAGAACAAATGGAAGA CCTTTAAACACTGAAAACGGATCCAGTGACTCATCCCGATTCTTGCACA
		site 3 mutant	GGGACTTGGGTGACTCTAGGGCACTGGTATGTCACAAAGCTGGTGACTCAGC CTTTAAACACTGAAAACGGGGAAGAACGTTAGATTTAGTTCTTGCACA
		all sites mutant	GGGACTTGGGGCGGAGAAGGGCACTGGTATGTCACAAGAACAAATGGAAGA CCTTTAAACACTGAAAACGGGGAAGAACGTTAGATTTAGTTCTTGCACA

Table S5. Correlations between miR-5188 expression and the clinicopathological features of hepatocellular carcinoma patients

Characteristics	n	miR-5188 expression		*P* value
		Low	High	
Age (years)				
≤Median	91	42 (46.2%)	49 (53.8%)	0.185
＞Median	93	52 (56.4%)	41 (43.6%)	
Gender				
Male	159	77 (48.4%)	82 (51.6%)	0.049
Female	26	18 (69.2%)	8 (30.8%)	
AJCC stage				
Ⅰ-Ⅱ	133	77 (57.9%)	56 (42.1%)	0.026
Ⅲ-Ⅳ	44	17 (38.6%)	27 (61.4%)	
T classfication				
T1-T2	142	79 (55.6%)	63 (44.4%)	0.034
T3-T4	43	16 (37.2%)	27 (62.8%)	
N classfication				
N0	176	93 (52.8%)	83 (100.0%)	1.000
N1	1	1 (47.2%)	0 (0.0%)	
Distant metastasis				
No	177	95 (53.7%)	82 (46.3%)	0.466
Yes	1	0 (0.0%)	1 (100.0%)	
Vascular invasion				
No	144	69 (47.9%)	75 (52.1%)	0.080
Yes	41	26 (63.4%)	15 (36.6%)	
Hepatic cirrhosis				
No	66	30 (45.5%)	36 (54.5%)	0.232
Yes	119	65 (54.6%)	54 (45.4%)	
HBsAg				
Negative	13	11 (84.6%)	2 (15.4%)	0.047
Positive	81	45 (55.6%)	36 (44.4%)	
Recurrence				
No	37	28 (75.7%)	9 (24.3%)	0.028
Yes	53	28 (52.8%)	25 (47.2%)	
AFP (ng/ml)				
≤20	27	17 (63.0%)	10 (37.0%)	0.924
＞20	63	39 (61.9%)	24 (38.1%)	
Total bilirubin (umol/L)				
≤20	74	44 (59.5%)	30 (40.5%)	0.245

＞20	16	12 (75.0%)	4 (25.0%)	
ALT (U/L)				
≤45	50	32 (64.0%)	18 (36.0%)	0.697
＞45	40	24 (60.0%)	16 (40.0%)	
GGT (U/L)				
≤40	25	17 (68.0%)	8 (32.0%)	0.483
＞40	65	39 (60.0%)	26 (40.0%)	
Edmondson-Steiner grade				
Ⅰ-Ⅱ	61	20 (32.8%)	41 (67.2%)	0.414
Ⅲ-Ⅳ	34	14 (41.2%)	20 (58.8%)	
Tumor number				
Single	25	9 (34.0%)	16 (64.0%)	0.539
Multiple	45	13 (29.5%)	32 (70.5%)	
Tumor size (cm)				
≤5	42	13 (31.0%)	29 (69.0%)	0.344
＞5	52	21 (40.4%)	31 (59.6%)	

Table S6. Univariate and multivariate survival analysis of clinicpathological variables of hepatocellular carcinoma patients

	Overall survival					
	univariate analysis			Multivariate analysis		
**Characteristics**	**HR**	**95% CI**	***P* value**	**HR**	**95% CI**	***P* value**
miR-5188 expression	0.59	(0.403-0.864)	0.007	0.684	(0.482-0.970)	0.033
Low *vs*. High						
Age (years)	0.927	(0.239-1.615)	0.865			
≤Median *vs*. ＞Median						
Gender	1.575	(0.956-2.595)	0.074			
Male *vs*. Female						
AJCC stage	0.256	(0.150-0.436)	< 0.001	0.509	(0.322-0.805)	0.004
Ⅰ-Ⅱ *vs*. Ⅲ-Ⅳ						
T classfication	0.286	(0.170-0.481)	< 0.001	0.598	(0.385-0.930)	0.022
T1-T2 *vs*. T3-T4						
N classfication	0.045	(0.001-2.418)	0.127			
N0 *vs*. N1						
Distant metastasis	0.000	(0.000-0.000)	< 0.001			
No *vs*.Yes						
Recurrence	0.217	(0.123-0.385)	< 0.001	0.131	(0.058-0.296)	< 0.001
No *vs*. Yes						
HBsAg	1.222	(0.543-2.754)	0.628			
Negative *vs*. Positive						
Vascular invasion	0.508	(0.313-0.822)	0.006	0.564	(0.385-0.827)	0.003
No *vs*. Yes						
Hepatic cirrhosis	1.269	(0.849-1.897)	0.245			
No *vs*. Yes						
AFP (ng/ml)	0.872	(0.474-1.602)	0.658			
≤20 *vs*. ＞20						
Total bilirubin (umol/L)	1.453	(0.690-3.059)	0.325			
≤20 *vs*. ＞20						
ALT (U/L)	0.638	(0.357-1.138)	0.128			
≤45 *vs*. ＞45						
GGT (U/L)	0.643	(0.350-1.180)	0.154			
≤40 *vs*. ＞40						

Edmondson-Steiner grade	0.725	(0.421-1.249)	0.247			
Ⅰ-Ⅱ *vs.*Ⅲ-Ⅳ						
Tumor number	1.73	(0.896-3.339)	0.102			
Singlevs *vs. Multiple*						
Tumor size (cm)	0.475	(0.283-0.799)	0.005	0.966	(0.531-1.760)	0.911
≤5 *vs.* ＞5						
